# Long-acting granulocyte colony-stimulating factor in primary prophylaxis of early infection in patients with newly diagnosed multiple myeloma

**DOI:** 10.1007/s00520-022-06851-8

**Published:** 2022-01-22

**Authors:** Xinjing Ding, Jianghua Ding, Hong Gu, Chuanxiang Zhong

**Affiliations:** 1 Department of Undergraduate, The Medical College of Nanchang University, Nanchang, Jiangxi Province China; 2 Department of Hematology & Oncology, The Affiliated Hospital of Jiujiang University, No. 57#, Xunyang East Road, Jiujiang, Jiujiang City, Jiangxi Province China; 3 Department of Hematology & Oncology, The People Hospital of Ruichang, Ruichang, Jiangxi Province China; 4 Department of Hematology & Oncology, The No. 171 Hospital of PLA, Jiujiang, Jiangxi Province China

**Keywords:** Long-acting G-CSF, Primary prophylaxis, Early infection, Newly diagnosed multiple myeloma

## Abstract

**Purpose:**

This study sought to compare the efficacy of prophylactic long-acting and standard granulocyte colony-stimulating factor (G-CSF) on febrile neutropenia, early infections, and treatment delay in patients with newly diagnosed multiple myeloma (MM) receiving the therapeutic regimen of bortezomib, lenalidomide, and dexamethasone (VRd).

**Methods:**

A prospective study with 68 consecutive patients with MM was conducted in three regional hospitals. Participants were randomly treated with the VRd regimen in combination with prophylactic long-acting G-CSF (treatment group) or prophylactic standard G-CSF (control group). The primary endpoints were the incidence rates of febrile neutropenia, early infection, and treatment delays. The secondary endpoint was clinical outcomes.

**Results:**

Thirty-three patients were assigned to the treatment group, and thirty-five patients were assigned to the control group. The incidence of febrile neutropenia was 6.1% and 17.1% in the treatment and control groups, respectively (*p* = 0.297). However, the rates of early infection and treatment delay were markedly lower in the treatment group than in the control group (6.1% vs. 25.7% and 9.1% vs. 31.4%; *p* < 0.05). Notably, all early infections occurred during the first four cycles of VRd therapy, and the most common type of infection was pneumonia. No significant difference in clinical efficacy was found between the two groups. All participants achieved at least partial remission.

**Conclusions:**

Prophylactic administration of domestic long-acting G-CSF markedly reduced the rates of early infection and treatment delay as compared with standard G-CSF in patients newly diagnosed with MM. Notably, all early infections occurred during the first four cycles of VRd therapy. As such, it seems appropriate to administer long-acting G-CSF with the aim of primary prophylaxis of early infection in the setting of newly diagnosed MM.

## Introduction

Patients with multiple myeloma (MM) experience an increased chance of infection than the general population, with the risk being 13 times more than that for pneumonia and 30 times more than that for sepsis [1, 2]. The greater risk of infection in patients with MM can be attributed to advanced age; disease-related immunodeficiency involving humoral immunity and treatment-related toxicity, particularly for myelotoxicity; and comorbidities such as diabetes and chronic obstructive pulmonary diseases. Notably, the infection itself also contributes to the progression of MM via the production of interleukin-6 and the activation of Toll-like receptors on myeloma cells [3]. Importantly, infection is the leading cause of morbidity and mortality among patients with MM, with around 45% of deaths in this population resulting from infection [2]. Currently, with the continual advent of several novel anti-myeloma drugs, the 5-year survival rate of patients with MM has risen to about 60% [4]. Therefore, it is of paramount importance to continue efforts to prevent deaths from infection among these individuals.

The entry of infectious organisms, including bacteria and viruses, into the body can occur during any treatment phase in patients with MM. The result of the FIRST study, which examined the prevalence of infections among patients with MM, showed that the infection rate was 56.2% over the initial four cycles of the treatment period. In particular, 30% of infections occurred in the first month of treatment [5]. So, more attention should be paid to the prevention of infection during the first and second courses of treatment in patients with MM. As well known, the most common therapy for bacterial infections is the use of antibiotics. However, European Organization for Research and Treatment of Cancer (EORTC) guideline does not recommend universal antibacterial prophylaxis in patients with hematological and solid tumors, including those with MM [6, 7].

In patients with MM, neutropenia not only causes treatment delays or dose reductions but is also seen as an independent risk factor for a poor prognosis [3]. The national cancer center network (NCCN) guideline on managing febrile neutropenia (FN) recommends prophylactic treatment of granulocyte colony–stimulating factor (G-CSF) for patients receiving chemotherapy and at high risk of more than 20% of developing FN. For patients newly diagnosed with MM, up to 56.2% experienced infections during their initial four courses of chemotherapy [5]. Importantly, there is a direct correlation between a lower neutrophil cell count and a greater risk of infection (mainly bacterial and fungal infections) in patients with MM [8]. Therefore, it is reasonable to adopt prophylactic use of G-CSF for infection in patients with MM with neutropenia.

In recent years, long-acting G-CSF therapies, such as pegfilgrastim and biosimilar drugs, have been widely applied in cancer-related supportive care due to their higher efficacy and better adherence. The vast majority of clinical studies have focused on the mobilization outcome of pegfilgrastim in autologous stem cell transplantation of MM [9]. In contrast, only a few studies (including just three clinical trials and three observational studies) have considered primary prophylaxis with pegfilgrastim for FN in refractory and relapsed patients with MM who received lenalidomide- or bendamustine-based regimens [10–15]. These studies reported the supportive role of pegfilgrastim in ensuring scheduled chemotherapy and decreasing the rates of neutropenia and infection; however, all investigations were both single-armed or retrospective studies and only enrolled patients with recurrent or refractory MM. As mentioned above, the risk of infection is highest during the first 4 months of treatment, owing to the huge tumor burden and frail physical status typically present in patients with MM [5]. In the present study, we included patients newly diagnosed with MM, aiming to compare the primary prophylactic efficacy of early infection using long-acting and standard G-CSF. The primary endpoints were the incidence rates of early infection and treatment delays in patients newly diagnosed with MM.

## Methods

### Patients

A total of 68 consecutive patients with MM were included from March 1, 2019, to July 20, 2021, at three regional hospitals. The eligibility criteria for inclusion in this study were as follows: (1) newly diagnosed with systematic MM; (2) aged 18–75 years; (3) Eastern Cooperative Oncology Group (ECOG) score of less than three points; and (4) no history of cardiac arrhythmias, congestive heart failure, or severe coronary artery disease.

All patients received anti-myeloma therapy, except autologous hematopoietic stem cell transplantation. The last study follow-up date was June 30, 2021. Patients with amyloidosis or without a willingness to receive treatment were excluded.

### Treatment protocol

According to medication use of long-acting or standard G-CSF, all patients were randomly divided into two groups of 33 patients treated with the chemotherapy with long-acting G-CSF (treatment group) and 35 patients treated with the chemotherapy with standard G-CSF (control group).

The specific chemotherapy protocol, known as the VRd regimen, was as follows: 1.3 mg/sqm of bortezomib (Qilu-pharma, Jinan, China) on days 1, 4, 8, and 11; 20–25 mg/d of lenalidomide (SL Pharm, Beijing, China) for 14 days; and 20 mg/d of dexamethasone on days 1, 2, 4, 5, 8, 9, 11, and 12. According to the regimens described in the literature by Cerchione et al. [13], the patients in the treatment group were given long-acting G-CSF (6 mg) (Xinruibai, Qilu-pharma, Jinan, China) subcutaneously with a single administration performed on the 6th day as primary prophylaxis, while the patients in the control group were treated by primary prophylaxis of standard G-CSF (5 μg/kg/day on days 6–8 of each cycle) (Ruibai, Qilu-pharma, Jinan, China). For the purposes of this study, a treatment cycle was 21 days in length, and all patients underwent 6–8 courses of chemotherapy and then continued to receive lenalidomide therapy until an unacceptable adverse event or disease progression occurred. Lenalidomide was given orally once daily with the starting dose was 25 mg/day for all patients. The dose reduced to 20 mg/day in patients who experienced febrile neutropenia or grade 4 thrombocytopenia. Blood routine, liver and kidney functions, C-reactive protein level, immunoglobulin concentration, and serum-free light chain results were examined before and after chemotherapy of each cycle.

### Study endpoints

The primary endpoints were the incidence rates of FN, early infection, and treatment delay. An episode of FN was defined as follows: (1) fever, a single axillary temperature greater than 38.0 °C or an axillary temperature greater than 37.5 °C lasting 1 h and (2) neutropenia, defined according to the FN guidelines from NCCN [16]. The criteria for infection in this study were defined as the existence of a pathogen and imaging evidence of infection combined with concomitant clinical symptoms, such as non-pharmacological rise in body temperature (> 37 °C), cough with sputum, and painful urination. Bacteria and fungi were identified by morphological, biochemical, and serological reactions after isolation, purification, and cultivation [17]. Early infection was deemed as that occurring during the first 4 months of treatment [5]. Treatment delay referred to chemotherapy administration occurring more than 2 days after the scheduled date. The secondary endpoint was patient clinical outcomes.

Efficacy was assessed every two cycles of treatment according to the new categories from the International Myeloma Working Group, which include stringent complete remission (sCR), complete remission (CR), very good partial remission (VGPR), partial remission (PR), minimal response (MR), stable disease, and progressive disease [18].

### Statistical analysis

All statistical analyses were conducted using the Statistical Package for the Social Sciences software program (version 26; IBM Corporation, Armonk, NY, USA). An independent *t*-test was run to examine the differences between the two groups. Fisher’s exact test and a chi-squared test were employed to compare the binary outcomes. The degree of clinical efficacy was analyzed by Ridit analysis. A *p*-value less than 0.05 was considered to be statistically significant, based on a two-sided test.

## Results

### Clinical characteristics

A total of 68 patients with MM were enrolled in this study, including 33 and 35 patients assigned to the treatment and control groups, respectively. As shown in Table 1, the two groups were very similar in terms of baseline characteristics, including age, sex, Revised International Staging System (R-ISS) stage, ECOG score, and cytogenetic abnormalities (*p* > 0.05).

**Table 1:** Comparison of clinical characteristics between the treatment and control groups

Characteristic	Treatment group, *n* (%)	Control group, *n* (%)	*p*-value
Number	33	35	-
Age, years			
Median (range)	62 (54–71)	65 (56–75)	0.3538
Sex			
Male	18 (54.6%)	16 (45.7%)	
Female	15 (45.4%)	19 (54.3%)	0.4666
R-ISS stage			
I–II	16 (48.5%)	15 (42.8%)	
III	17 (51.5%)	20 (57.1%)	0.9253
ECOG			
0–1	28 (84.8%)	27 (77.1%)	
≥ 2	5 (15.2%)	8 (22.9%)	0.4193
Cytogenetic abnormality			
Standard risk	13 (39.4%)	18 (51.4%)	
High risk	20 (60.6%)	17 (48.6%)	0.3193

An independent *t*-test was run to examine the differences of ages from two groups. Fisher’s exact test and a chi-squared test were used to compare the other binary outcomes.

### Incidence rates of FN, early infection, and treatment delay

Throughout the period of chemotherapy, 6.1% and 17.1% of cases of FN were detected in the treatment and control groups, respectively, but there was no statistical difference between the two groups. However, as shown in Table 2, we discovered that the rates of early infection and treatment delay in the treatment group were markedly lower than those in the control group. As shown in Table 3, all early infections occurred during the initial second to fourth cycles. There were 6.1% episodes of dose adjustment in the treatment group and 17.1% episodes in the control patients. No grade 4 thrombocytopenia was observed in the two groups.

**Table 2:** Clinical events between the treatment and control groups

Event	Treatment group (*n* = 33), *n* (%)	Control group (*n* = 35), *n* (%)	*p*-value
Febrile neutropenia	2 (6.1%)	6 (17.1%)	0.297
Early infection	2 (6.1%)	9 (25.7%)	0.0278^#^
Treatment delay	3 (9.1%)	11 (31.4%)	0.0228^#^
Dose adjustment	2 (6.1%)	6 (17.1%)	0.297

*Fisher*’s exact test and a chi-squared test were performed to compare the binary outcomes.

^#^The differences were statistically significant.

**Table 3:** Comparison of early infections in patients between the treatment and control groups

Type of infection	Treatment group (*n* = 33), *n*	Control group (*n* = 35), *n*	Cycle sequence(s) of infection occurrence
Pneumonia	1	5	2–4
Colitis	0	2	2–3
Fever of unknown origin	1	2	2–4
Total	2	9	

### Clinical outcomes

All 68 patients completed at least six cycles of chemotherapy, and 60 (88.2%) patients completed the prescribed eight cycles. As shown in Table 4, there were not marked differences in clinical outcomes between the two groups. No stringent complete remission efficacy was found in either group; however, all patients achieved at least PR of their disease.

**Table 4:** Clinical outcomes between the treatment and control groups

Efficacy	Treatment group (*n* = 33), *n* (%)	Control group (*n* = 35), *n* (%)	*p*-value
sCR	0 (0.0%)	0 (0.0%)	0.2305
CR	3 (9.1%)	1 (2.9%)	
VGPR	23 (69.7%)	22 (62.9%)	
PR	7 (21.2%)	12 (34.3%)	
MR	0 (0.0%)	0 (0.0%)	

*sCR* Stringent complete remission; *CR* complete remission; *VGPR* very good partial response; *PR* partial remission; *MR* minimal response.

The degree of clinical efficacy was compared by *Ridit* analysis.

## Discussion

In the era of emerging novel treatments, the clinical outcomes of patients with MM have improved greatly to a 5-year survival rate of 60% [4]. However, MM remains notorious for placing patients at high risk of infection, thanks to the impairment of host defenses, and ensuring treatment-related immunosuppression. Importantly, the risk of infection is the highest within the first 4 months after the initial diagnosis. Furthermore, infection contributes to approximately 45% of early deaths [2]. Although the transition from chemotherapy to novel agents has caused a marked decrease in the incidence of severe pneumonia, heightened rates of early infection and pneumonia persist. Nearly all recent studies investigating MM-related infection focused on patients with refractory and relapsed MM [10–15]. As compared with these patients, those newly diagnosed with MM also experience the same risk of infection [19]. Therefore, it is imperative to prevent the occurrence of infection in all patients with MM, particularly early infection within the first 4 months after diagnosis.

As the vast majority of infections are bacterial in nature, it is reasonable, logically, to administer antibiotics for the prevention of infection in MM patients. A phase III study explored the effects of oral antibiotic prophylaxis, i.e., ciprofloxacin or trimethoprim-sulfamethoxazole, on the incidence of infection in patients with newly diagnosed MM; however, the study revealed that routine use of prophylactic antibiotics did not reduce the incidence of infection [7]. On the contrary, the other two studies supported the prophylactic use of levofloxacin in MM cases due to the marked decrease in severe infection observed relative to the control group (30.9% vs. 17.5%, *p* = 0.037; 27% vs 19%, *p* = 0.0018) [20, 21]. However, about 50% of patients withdrew from the TEAMM study due to severe infection [21]. Moreover, prophylactic antibiotics can also increase the risk of *Clostridioides difficile* infection and antibiotic resistance. The discrepancy was largely attributed to significant differences in therapeutic regimens and enrolled patients with MM. Currently, prophylactic antibiotics for early infection should be limited to use in high-risk patients who have the heightened potential to experience severe-grade and prolonged neutropenia during anti-myeloma treatment [22].

In the clinical setting, neutropenia has been strongly associated with the incidence of infection in patients with MM [8, 23]. Owing to the fragile performance status and multiple comorbidities (e.g., diabetes) of patients with MM, neutropenia-related infection may be a potentially life-threatening condition in such patients, which also results in treatment delay and the final poor outcome. Thus, the idea of G-CSF as a feasible treatment option has emerged to prevent neutropenia-related infections in those with MM. As noted previously, long-acting G-CSF has been documented for prophylaxis of infection mainly in refractory and relapsed patients with MM [10–15]. Enlightened by such previous findings, we investigated the effect of long-acting G-CSF on preventing infection among those with newly diagnosed MM.

In our study, the incidence rates of early infection and treatment delay were markedly lower in the long-acting G-CSF group than in the standard G-CSF group. The underlying mechanisms were attributed to the longer circulation half-life of long-acting G-CSF in comparison with standard G-CSF [24]. We detected a tendency for reduction of FN in the long-acting G-CSF in compared with standard G-CSF group (6.1% vs. 17.1%; *p* = 0.297), but there was no significant difference, which might be ascribed to the relatively small sample sizes. Further analysis reported that the common types of early infection included pneumonia, colitis, and fever of unknown origin. Of note, pneumonia was the most frequent type of infection, accounting for more than 50% of early infections in our study. This finding was consistent with the results of other research, such as that by Lavi et al. [25], who strongly recommended chest computed tomography as a routine examination for patients with MM at high risk of infection. In the present study, the VRd regimen was chosen as the front induction therapy due to the reported pronounced improvements in overall response rate, progression-free survival, and overall survival, especially among patients with high-risk features [26]. As for clinical efficacy, there were no significant differences between the long-acting and standard G-CSF groups. All of our participants achieved PR or better outcomes following the VRd plus G-CSF regimen.

## Conclusions

For patients newly diagnosed with MM, the results of this study revealed that domestic long-acting G-CSF administered on day 6 led to marked decreases in early infection and treatment delay relative to standard G-CSF, while no difference was detected between these two regimens in terms of the incidence of FN. Moreover, all of the early infections in this study occurred during the initial second to fourth cycles of the VRd regimen, and the most common type of early infection was pneumonia. In light of these results, it appears appropriate to administer long-acting G-CSF for the prevention of early infection. However, there were some deficiencies in this study. One was its small sample size, and another was the single kind of standard treatment protocol employed herein. Future clinical studies with larger sample sizes are necessary to confirm our results.

## Data Availability

Not applicable.
